# Level-dependent IMD cancellation for single-transducer DPOAE measurement using a lookup-table method

**DOI:** 10.1016/j.mex.2026.104008

**Published:** 2026-06-14

**Authors:** Irwansyah, Sho Otsuka, Seiji Nakagawa

**Affiliations:** a Center for Frontier Medical Engineering, Chiba University, 1-33 Yayoi-cho, Chiba, Inage-ku 263-8522, Japan; b Graduate School of Engineering, Chiba University, 1-33 Yayoi-cho, Chiba, Inage-ku 263-8522, Japan; c Med-Tech Link Center, Chiba University Hospital, 1-8-1 Inohana, Chuo-ku, Chiba, Chiba-shi 260-8677, Japan

**Keywords:** Otoacoustic emissions, Intermodulation distortion, Ear-canal sound pressure, Lock-in amplifier technique, Single-transducer stimulation, Probe microphone

## Abstract

Distortion product otoacoustic emissions (DPOAEs) are weak cochlear tones (*f*_3_ = 2*f*_1_ − *f*_2_) evoked by two primary tones (*f*_1_, *f*_2_) and used clinically to assess cochlear function. In practice, *f*_1_ and *f*_2_ are typically presented using two independent sound sources. This is because using one transducer to output both tones can generate intermodulation distortion (IMD) at the same frequency (*f*_3_) as the DPOAE due to transducer nonlinearity, which makes it difficult to distinguish measurement artifacts from biological emissions. As a result, DPOAE measurement is difficult with conventional earphones, which typically use one playback transducer per ear. Here, we describe a method that enables single-transducer DPOAE measurements by adding a cancellation tone at *f*_3_ and tuning its amplitude and phase (via a lock-in amplifier technique) to reduce IMD. Since IMD is level dependent and in-ear stimulus levels vary across ears (due to ear-canal acoustics), the required cancellation parameters are therefore stored in a level-specific lookup table (LUT). The proposed method:

• Reduces IMD at the DPOAE frequency to enable single-transducer measurement.
• Uses LUT parameters to handle level-dependent IMD.
• Was validated on 40 ears against a two-transducer baseline.

## Graphical abstract

**Figure:**
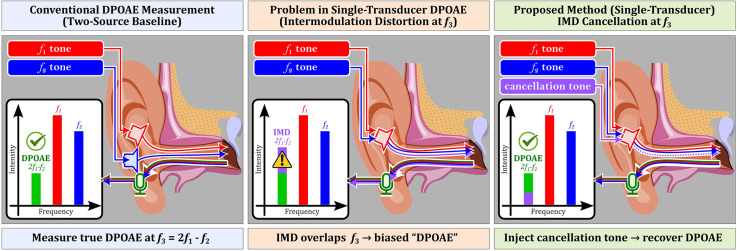

## Specifications table

**Table:** 

Subject area	Medicine and Dentistry
More specific subject area	Audiology
Name of your method	LUT-Based IMD Cancellation for Single-Transducer DPOAE Measurement
Name and reference of original method	Irwansyah, S. Otsuka, and S. Nakagawa, “DPOAE Responses in Humans and Ear Models: Can a Single-Loudspeaker Approach Match the Two-Loudspeaker Technique?”, in IEEE Access, 13 (2025) 103,095–103,108. https://doi.org/10.1109/ACCESS.2025.3579475
Resource availability	N.A.

## Background

Distortion-product otoacoustic emissions (DPOAEs) are weak acoustic responses (typically below or around 20 dB SPL in the ear canal) generated in the cochlea when two primary tones (*f*_1_, *f*_2_) are presented [[1], [2], [3]]. DPOAEs are widely used in clinical and research settings as an objective measure of outer hair cell function [4,5]. The most analyzed component in clinical practice appears at 2*f*_1_ − *f*_2_ and is recorded in the ear canal while the primary tones are presented using a probe system [[6], [7], [8]]. Recently, there has been growing interest in implementing OAE measurements, including DPOAE, using conventional earphones and earbuds, such as consumer in-ear devices that already include microphones and digital signal processing [[9], [10], [11]]. However, conventional DPOAE probes typically require two independent sources to present *f*_1_ and *f*_2_ tones, while conventional earphones often only have a single playback transducer per ear [[11], [12], [13]]. Thus, these configuration differences motivate the investigation of single-transducer DPOAE measurement methods for potential earphone-based implementation.

**Fig. 1:**
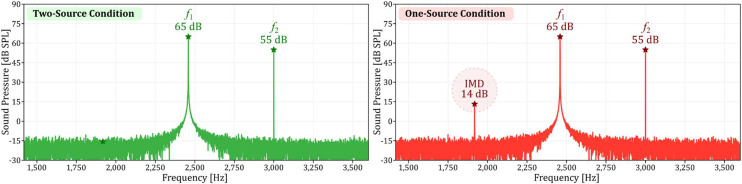
Example spectra measured in an ear simulator. Primary tones were *f*_2_ = 3000 Hz with *f*_2_ / *f*_1_ = 1.22 (*L*_1_, *L*_2_ = 65, 55 dB SPL). IMD component is absent at *f*_3_ in the two-source condition (left) but present in the one-source condition at 14 dB SPL (right).

One practical challenge with single-transducer configurations is the transducer nonlinearity itself [12]. When a single transducer outputs both primary tones, *f*_1_ and *f*_2_, intermodulation distortion (IMD) can produce a component at *f*_3_=2*f*_1_ − *f*_2_ [[11], [12], [13], [14]]. IMD here refers to transducer-generated distortion. Since this is the same frequency used to detect DPOAE, IMD artifacts overlap with biological emissions [12]. As a result, the emissions measured at *f*_3_ in the ear canal may contain both biological emissions and transducer-generated distortion components, making separation difficult [12,14]. This issue can be easily illustrated using an ear model that does not contain biological contributions (i.e., produces no DPOAE). Fig. 1 compares two-source and single-source stimulation in an IEC 60318–4 ear simulator [15]. The 2*f*_1_ − *f*_2_ component is measured in the single-source condition, but is absent in the two-source condition.

**Fig. 2:**
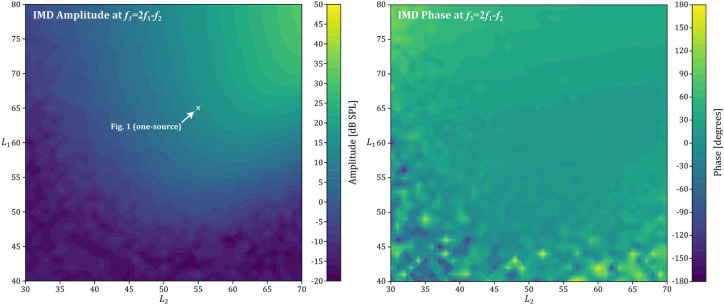
IMD maps at *f*_3_=2*f*_1_ − *f*_2_ in the one-source condition (IEC 60318–4 ear simulator; *f*_2_ = 3000 Hz, *f*_2_ / *f*_1_ = 1.22): amplitude (left) and phase (right) as a function of primary levels *L*_1_ and *L*_2_. The “x” marks the operating point used for Fig. 1 (one-source).

To enable single-transducer DPOAE measurement, several techniques have been reported to suppress IMD at *f*_3_ [11−14]. These include techniques based on distortion cancellation [12,14] and nonlinear modeling [11]. These techniques rely on estimating the complex IMD components at *f*_3_, i.e., magnitude (amplitude) and phase, to effectively suppress IMD at a specific operating point (*L*_1_/*L*_2_ = 65/55 dB SPL). Once these parameters are known, the IMD at *f*_3_ can be suppressed to a sufficiently low level [12,14]. However, in actual measurements, the level (*L*_1_ or *L*_2_) obtained in the ear canal often does not match the intended level [16,17]. This is due to variations in ear canal acoustics and earphone/probe fit between individuals [17]. Therefore, in-ear level calibration and adjustment are typically performed [18,19]. As a result, the operating point (*L*_1_, *L*_2_) may vary from ear to ear. Since IMD itself is a function of *L*_1_ and *L*_2_, the cancellation parameters may not be optimal. To demonstrate this level dependency, we measured the IMD level and phase using an ear simulator and mapped them as shown in Fig. 2. Furthermore, to enable single-transducer measurements under such level-dependent condition (see Fig. 2), this method article describes a level-dependent implementation in which cancellation parameters are organized as a lookup table (LUT) indexed by *L*_1_ and *L*_2_. After applying the selected in-ear calibration method, the corresponding cancellation tone can be selected from the LUT.

## Method details

### Overview of the lookup-table workflow

The method we describe in this paper is intended to create a level-specific look-up table (LUT), that is, a table containing the amplitudes and phases of the cancelling tones required to suppress IMD at frequency *f*_3_ in a single-transducer configuration. In this method, the LUT is defined based on the sound pressure level (SPL) measured by the probe microphone. Therefore, the indices on the LUT represent the actual primary levels (*L*_1_, *L*_2_), not the electrical drive levels. This allows the LUT to be used in conjunction with in-ear calibration, where *L*_1_ and *L*_2_ are adjusted to the target SPL based on the probe microphone measurements.

To construct and implement the LUT, our method relies on a comparison of the acoustic frequency response measured in an ear simulator with that of the human ear. In the ear simulator, a time-stretched pulse (TSP) signal is played to estimate the frequency response between the earphone and the probe microphone. Next, the IMD cancellation parameters are measured and estimated at various combinations (*L*_1_, *L*_2_), and then stored in the LUT. In real-ear measurements, the same TSP procedure is used to estimate the frequency response of the ear canal. The difference between the two responses is then used to correct or select appropriate LUT-based cancellation parameters for each ear. An example of this process is shown at the end of this section.

### Equipment and measurement setup

**Fig. 3:**
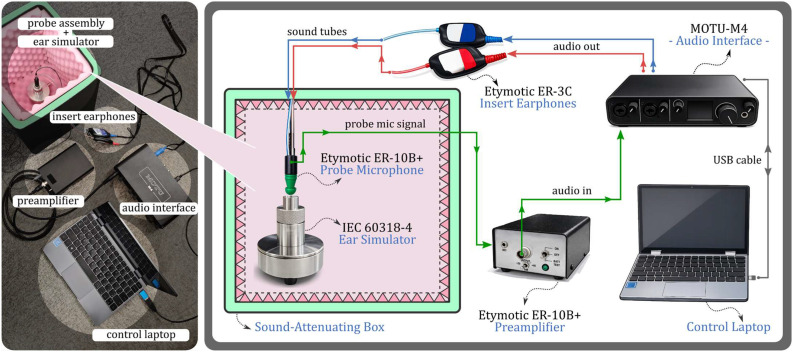
Measurement setup and signal flow for playback and probe-microphone recording in an IEC 60318–4 ear simulator using insert earphones, ER-10B+ probe microphone/preamplifier, and an audio interface controlled from a laptop.

Table 1 summarizes the equipment used in the measurement setup. The setup is centered on an insert earphone for stimulus delivery and a probe microphone system for recording sound pressure inside an IEC 60318–4 ear simulator. Playback and recording processes are controlled from a laptop via an audio interface, and the recorded signals by the probe microphone are then used for subsequent analysis. Measurements should be performed in an acoustically quiet environment to reduce contamination from external noise. An anechoic chamber is ideal, but not mandatory. In our implementation, measurements were performed in a normal room, with the ear simulator and probe assembly placed inside a small, sound-attenuating box (3D-printed and lined with ∼1-cm silicone rubber) to attenuate external noise during recording. The overall measurement setup and signal flow are shown in Fig. 3.

**Table 1:** Equipment list (recommended vs. used).

Type	Recommended	Used in this study
Ear simulator	IEC 60318–4 (or equivalent occluded-ear simulator)	IEC 60318–4 ear simulator
Probe microphone system	Low-noise probe microphone system with preamplifier (for OAE/DPOAE recording)	ER-10B+ probe microphone system + preamplifier
Insert earphone	Insert earphones suitable for in-ear acoustic stimulation	ER-3C insert earphones
Audio interface	Interface with synchronized playback and recording (single clock / same interface)	MOTU-M4
Control computer	Any PC/laptop that can be used for stimulus control and data acquisition	Laptop with Linux OS
SPL reference for mic check	Reference tone / calibrator procedure (commonly 1 kHz)	ND9B sound level calibrator (1 kHz)
Acoustic environment	Quiet environment (anechoic room if available)	Normal room + small sound-attenuating box

### Step-by-step procedure for building and using the IMD-cancellation lookup table

The following steps present a replicable workflow for constructing an IMD-cancellation lookup table (LUT) and using it in a single-transducer configuration. First, the sensitivity of the probe microphone is calibrated, and then an IEC 60318–4 ear simulator system is assembled. The transfer response between the earphone and the probe microphone is then measured to establish and correct the stimulus level. Next, the LUT is then constructed by estimating the IMD component at *f*_3_ and calculating the amplitude and phase of the cancellation tone at various combinations of primary levels (*L*_1_, *L*_2_). Once the LUT is created in the ear simulator, the final step provides an example showing how a single LUT entry can be applied to a single ear canal load by adjusting the cancellation tone at *f*_3_ based on the difference in transfer response. Details for each step are provided below:

❖ Step 1—*Probe-microphone sensitivity calibration (1* *kHz sound-level calibrator)*

**Fig. 4:**
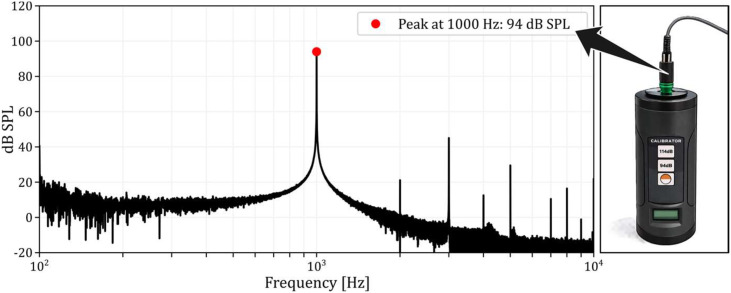
Representative FFT magnitude of the recording of the ND9B 1-kHz, 94-dB SPL tone measured with the probe microphone. The 1-kHz spectral peak was used to define the SPL calibration offset applied to subsequent measurements.

This step establishes the dB SPL scale for all subsequent measurements by calibrating the ER-10B+ probe microphone using a 1-kHz reference tone. We used an ND9B sound level calibrator, which produces a 1-kHz tone at 94 dB SPL. The probe microphone was connected to its preamplifier. Then, the signal was fed to the same audio interface (MOTU-M4) input as for the main measurements (Fig. 3), with the input gain fixed. The probe tip was inserted into the coupler opening on the calibrator that matched the probe diameter (Fig. 4, right panel). This was done to ensure a snug fit and a good seal. The sound level calibrator was then turned on and allowed to run until the tone stabilized. Once stable, we recorded the reference tone for about 10 s.

The raw recording was analyzed in Python. We applied the Fast Fourier Transform (FFT) and looked for the peak in the spectrum at 1 kHz. The measured 1-kHz peak magnitude, *A*_1kHz_ (in the “dB” scale used in subsequent spectrum plots), was mapped to 94 dB SPL with a single calibration offset: Δ = 94 dB SPL − *A*_1kHz_. This Δ value was added to all spectra measured with the same recording configuration and gain. Thus, each magnitude *A*(*f*) can be expressed in terms of SPL as *L*(*f*) = *A*(*f*) + Δ. This is because the probe microphone response was treated as nearly flat over the frequency range of 200 Hz to 10 kHz, as per the manufacturer’s calibration documentation. Note that this assumption may not hold for other probe microphone systems. When using a different probe microphone, the microphone transfer function should be measured or obtained from calibration data and applied as a frequency-dependent compensation in addition to the 1-kHz sensitivity calibration. The Δ value depends on the recording configuration. Therefore, Step 1 must be repeated if the probe microphone, preamplifier, audio interface input channel or gain setting are changed. As an example of this step, Fig. 4 (left panel) shows the FFT spectrum of the recorded 1-kHz calibration tone. We kept the same input channel and gain for all subsequent recordings.

❖ Step 2— *Assemble the ear-simulator measurement setup*

After the probe-microphone sensitivity was calibrated (Step 1), we assembled the measurement system as shown in Fig. 3 to record sound pressure inside the IEC 60318–4 ear simulator. The probe microphone was connected to its microphone preamplifier. The preamplifier output then fed into one of the audio interface input channels. The insert earphone for stimulus playback was driven from one of the audio interface output channels. The audio interface was connected to a control laptop, which performed synchronous playback and recording using Python.

The probe assembly was mounted on the ear simulator to ensure a stable and repeatable acoustic load. Two insert earphones were physically attached to the two sound tube ports on the probe assembly. During the LUT construction, however, only one earphone channel was used for playback (the single-source condition). The second earphone remained attached but was not driven. This earphone served only to “fill” the second port. All acoustic measurements were made using the signal from the probe microphone (not the ear simulator’s internal microphone), as the probe microphone served as the SPL reference used in subsequent steps. Measurements were conducted in a quiet room. During recording, the ear simulator and probe assembly were placed inside a small, sound-attenuating box (3D-printed and lined with approximately 1 cm of silicone rubber) to attenuate external noise.

❖ Step 3— *Measure the earphone–simulator transfer response and define the level-setting correction*

This step measures the earphone-simulator response at the probe microphone. The results are used to create a frequency-dependent level correction. This correction is then used to adjust the pure tone level in later steps. With the setup assembled as in Step 2, we first adjusted the earphone playback scale using a 1 kHz tone until the probe microphone's FFT peak at 1 kHz read 65 dB SPL, using the calibration offset obtained in Step 1.

**Fig. 5:**
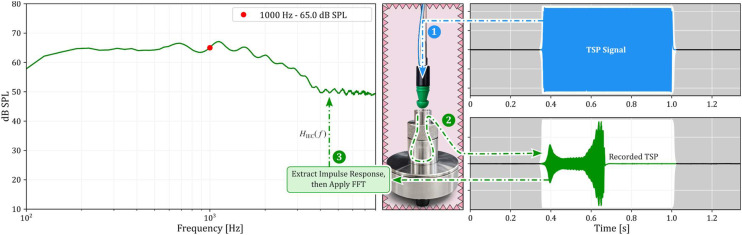
Measurement of the earphone–ear-simulator frequency response. (1) A TSP signal is played through the earphone into the ear simulator. (2) The probe microphone records the signal. (3) The impulse response is extracted and converted to the frequency-response magnitude *H*_IEC_(*f*), expressed in dB SPL; the 1-kHz reference point is indicated.

Using the same playback scale, we then played back the time-stretched pulse (TSP, #1 in Fig. 5) signal and recorded the TSP from the probe microphone. In this study, the specific TSP signal was an optimized Aoshima time-stretched pulse (OATSP), generated following Suzuki et al. [20]. This signal is different from a conventional swept-sine or chirp stimulus; it was not specified by explicit start and end frequencies or by onset/offset ramps. Instead, the OATSP was generated as a broadband discrete-time signal whose frequency-domain representation spans the positive-frequency range up to the Nyquist frequency. In practice, the usable frequency range was limited by the acoustic response of the earphone, probe microphone, and recording system. In our implementation, the signal length was *N* = 2*^i^* samples (*i* = 15, *N* = 32,768, ∼0.683 s) at a sampling frequency of 48 kHz. An example Python implementation of the OATSP signal generation and inverse-filter deconvolution is available at: https://github.com/iru-one-syah/OATSP. The recorded TSP (#2 in Fig. 5) was analyzed in Python to estimate the impulse response by deconvolution. Specifically, the recorded signal was deconvolved using an inverse TSP (or equivalently a matched inverse filter). The result was the earphone-simulator impulse response. The FFT of this impulse response then provided the corresponding frequency response magnitude, *H*_IEC_(*f*), in dB SPL (#3 in Fig. 5). It should be noted that the use of TSP is not mandatory in this step. The frequency response can also be estimated using other standard excitation methods, such as a swept-sine/chirp signal (with deconvolution) [21,22], MLS/pseudorandom sequence [22], or stepped tones, as long as the resulting impulse/frequency response is consistent.

Based on this measured response, we define a frequency-dependent level-setting correction, with a reference value of 65 dB SPL: *C*(*f*) = 65 − *H*_IEC_(*f*). By this definition, *C*(*f*) ≈ 0 at 1 kHz. Meanwhile, frequencies with lower measured responses will result in a positive correction. For example, if *H*_IEC_(4 kHz) = 50 dB SPL, then *C*(4 kHz) = +15 dB. This correction curve is used for frequency compensation when generating pure tones. For a tone at frequency *f* with a target level *L*_target_ in the probe microphone (e.g., *L*_1_ at *f*_1_, *L*_2_ at *f*_2_, or *L*_3_ at *f*_3_), the electric drive level is increased by *C*(*f*) (in dB). The goal here is to ensure that the measured level in the probe microphone matches the desired target. If necessary, this correction can also be checked by playing several pure tones (e.g., from 1 to 8 kHz) using the corrected amplitude, and then ensuring that the FFT peak of the probe microphone is close to the specified target SPL.

❖ Step 4— *Estimate IMD at f*_3_ *and compute cancellation parameters at one operating point (L*_1_*, L*_2_*)*

**Fig. 6:**
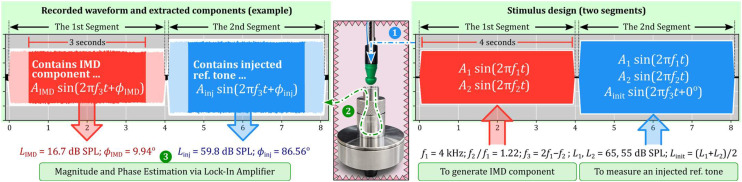
Two-segment stimulus and lock-in estimation used to measure the IMD component at *f*_3_ = 2*f*_1_−*f*_2_ and the injected reference tone at *f*_3_ in the ear simulator, and to compute the cancellation-tone level and phase for a given operating point (*L*_1_,*L*_2_).

This step estimates the IMD level and phase at *f*_3_ = 2*f*_1_−*f*_2_ for a single operating point (*L*_1_, *L*_2_). This step then calculates the cancelling tone parameters (level and phase at *f*_3_). As shown in Fig. 6 (right panel), we used a stimulus consisting of two segments. The first segment contained only the primary tones, whereas the second segment repeated the same primary tones and added a reference tone at *f*_3_. These two segments can be written as

(1a) $$s_{1}\left(t\right)=A_{1}\sin\left(2\pi f_{1}t\right)+A_{2}\sin\left(2\pi f_{2}t\right)$$

(1b) $$s_{2}\left(t\right)=A_{1}\sin\left(2\pi f_{1}t\right)+A_{2}\sin\left(2\pi f_{2}t\right)+A_{\text{init}}\sin\left(2\pi f_{3}t+\phi_{\text{init}}\right)$$

where *A*_1_ and *A*_2_ are linear amplitudes selected to realize the target levels *L*_1_ and *L*_2_ in dB SPL (using the frequency-dependent correction from Step 3). The first segment (1a) generates IMD at *f*_3_, which can be expressed at the probe microphone as *A*_IMD_ sin(2π*f*_3_*t*+*ϕ*_IMD_) (Fig. 6, left panel). In the second segment (1b), *A*_init_ is an amplitude to realize the level *L*_init_ in dB SPL. In our implementation, *L*_init_ = (*L*_1_+*L*_2_)/2 with an initial phase *ϕ*_init_ = 0° and each segment duration was 4 s with 50-ms onset/offset ramps. The two segments (1a) and (1b) were played through a single earphone channel. For analysis, we used only the steady portion of each segment (3 s), excluding 0.5 s at the beginning and end. The frequencies used in this step followed the nominal DPOAE relationship, with (*f*_2_/*f*_1_ = 1.22) and (*f*_3_ = 2*f*_1_−*f*_2_). They were not further adjusted to force an integer number of periods within the 3-s analysis window.

The recorded waveform from the probe microphone was then analyzed to extract the *f*_3_ component from each segment, as shown in Fig. 6 (left panel). We estimated the level and phase at *f*_3_ using digital lock-in calculations [14]. The lock-in calculation was used here because the target frequency was known in advance, and only the complex component at *f*_3_ was needed. This allows direct estimation of both level and phase at the target frequency for generating a cancellation tone. If *f*_3_ coincides with the FFT bin and the same analysis window is used, an FFT-based complex estimation can be used and is expected to give essentially the same cancellation parameters. This assumes that the amplitude scaling and phase conventions are treated consistently. Thus, the advantage here is that we can obtain a direct and convenient amplitude/phase estimate at a single frequency at *f*_3_, rather than a fundamentally different spectral estimate. Let *x*[*n*] denote the recorded samples in the analysis window (length *N* samples, sampling rate *f*_s_). The in-phase and quadrature components at *f*_3_ were calculated using Eq. (2):

(2) $$I=\sum_{n=0}^{N-1}x\left[n\right]\cos\left(2\pi \frac{f_{3}}{f_{s}}n\right),\;Q=\sum_{n=0}^{N-1}x\left[n\right]\sin\left(2\pi \frac{f_{3}}{f_{s}}n\right).$$

The corresponding magnitude and phase were then obtained using Eq. (3):

(3) $$A=\frac{2}{N}\sqrt{I^{2}+Q^{2}},\;\phi =\text{atan}2\;\left(I,Q\right)$$

where phase is computed for the sine-form representation *A* sin(2π*f*_3_*t* + *ϕ*). When this lock-in calculation is applied to the first segment, the result is (*L*_IMD_, *ϕ*_IMD_), the IMD component at *f*_3_. When applied to the second segment, the result is (*L*_inj_, *ϕ*_inj_), the tone injected and observed at the probe microphone at *f*_3_ (Fig. 6, left panel). Note that *L*_IMD_ and *L*_inj_ are obtained by converting the estimated linear amplitudes *A*_IMD_ and *A*_inj_ to dB SPL.

Finally, the cancelling tone was defined so that its magnitude is the same as the IMD component, but its phase is opposite (shifted by 180°). Using the injected tone as the amplitude/phase reference at *f*_3_, the required cancellation setting was calculated using Eq. (4):

(4) $$L_{\text{canc}}=L_{\text{IMD}}+\left(L_{\text{init}}-L_{\text{inj}}\right),\;\phi_{\text{canc}}=\phi_{\text{IMD}}+\left(\phi_{\text{init}}-\phi_{\text{inj}}\right)+180^{\circ }$$

with *ϕ*_canc_ wrapped to the selected phase range (e.g., 0° to 360°). The obtained values (*L*_canc_, *ϕ*_canc_) were then stored as LUT entries for the operating points (*L*_1_, *L*_2_).

Note that an alternative implementation of the second segment (Fig. 6, right panel) is to provide only the reference tone at *f*_3_. This approach can also work. This relies on the playback chain behaving linearly when the tone is scaled to the desired cancellation level. For example, reducing the digital stimulus amplitude by a given amount is expected to reduce the acoustic output by the same amount while preserving the phase. During method development, we observed that this was not always sufficient, especially at high *L*_1_ and *L*_2_ levels. A reference tone measured alone and then scaled to the estimated IMD level could still leave residual IMD, probably due to slight level or phase differences under the actual multi-tone condition. Therefore, in this study, the reference tone was measured while the primary tones were also present. This means that the measured *f*_3_ component in the second segment can include both the injected reference tone and the IMD generated by the primary tones. However, *L*_init_ was chosen as (*L*_1_+*L*_2_)/2, which was significantly larger (at least 34 dB) than the expected amount of IMD in our measurements, so the measured *f*_3_ component was mainly dominated by the injected reference tone. This practical choice was based on empirical observations during method development, although the difference between the *f*_3_-only and multi-tone reference approaches was not systematically quantified in this study.

❖ Step 5— *Verify suppression and store performance metrics*

**Fig. 7:**
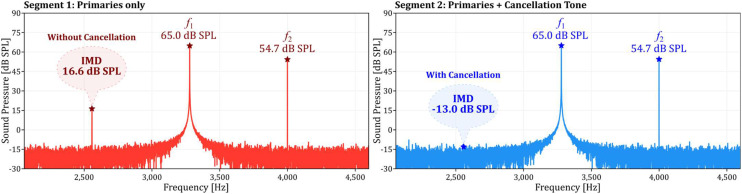
Example FFT spectra from the two-segment verification stimulus in the IEC 60318–4 ear simulator: Segment 1 contains only the primaries (*f*_1_, *f*_2_) and shows an IMD component at *f*_3_ (left), while Segment 2 adds the computed cancellation tone at *f*_3_ and reduces the IMD toward the noise floor (right).

This step aims to ensure that the cancellation parameters (*L*_canc_, *ϕ*_canc_) obtained in Step 4 effectively suppress the IMD at *f*_3_ for the same operating point (*L*_1_, *L*_2_). Using the same frequency pair (*f*_1_, *f*_2_), the same tone duration, and the same frequency-dependent level correction from Step 3, we created a two-segment verification stimulus again and recorded the probe microphone signal in the ear simulator. The two verification segments were the “without cancellation” segment *s*_0_(*t*) and the “with cancellation" segment *s*_c_(*t*), written as

(5a) $$s_{0}\left(t\right)=A_{1}\sin\left(2\pi f_{1}t\right)+A_{2}\sin\left(2\pi f_{2}t\right)$$

(5b) $$s_{c}\left(t\right)=A_{1}\sin\left(2\pi f_{1}t\right)+A_{2}\sin\left(2\pi f_{2}t\right)+A_{\text{canc}}\sin\left(2\pi f_{3}t+\phi_{\text{canc}}\right)$$

where *s*_0_(*t*) was used to measure the IMD level at *f*_3_ before applying cancellation, and *s*_c_(*t*) was used to verify the residual level after applying the cancellation tone. In Eq. (5b), *A*_canc_ and *ϕ*_canc_ were determined from the cancellation parameters (*L*_canc_, *ϕ*_canc_) obtained in Step 4. Here, the cancellation tone was scaled in linear amplitude *A*_canc_ to match the target *L*_canc_ in dB SPL. Fig. 7 shows representative spectra of these two segments in the ear simulator. In the segment containing only the primary tones (left panel), the IMD is visible at *f*_3_. In the segment with cancellation (right panel), on the other hand, the IMD drops near the noise floor. The spectra shown in Fig. 7 were calculated by applying a single FFT to the complete 3-s steady-state analysis window. The FFT length was equal to the number of samples in this interval, and no additional window function, zero padding, or time-domain averaging was applied. These spectra were used to visualize the IMD level before and after cancellation.

The IMD level before cancellation was stored as *L*_before_, which is obtained from the *f*_3_ peak in the “without cancellation” segment. The remaining level after cancellation was stored as *L*_after_, which is obtained from the *f*_3_ bin in the “with cancellation” segment. To assess whether the residual was close to the measurement floor limit, we also estimated the local noise floor around *f*_3_ of the “with cancellation” segment. We did this by averaging the spectral levels in the narrow bands just below and just above *f*_3_ (at *f*_3_ − Δ*f* and *f*_3_ + Δ*f*, excluding the *f*_3_ bin itself) such as ±20–50 Hz, and then storing the value as *L*_noise_. These three values {*L*_before_, *L*_after_, *L*_noise_} were stored for the current operating point (*L*_1_, *L*_2_), and were then used to visualize the suppression performance across the LUT grid (Step 6).

❖ Step 6— *Repeat across an (L*_1_*, L*_2_*) grid to populate the LUT*

This step builds the LUT by repeating the single operating point procedure (Steps 4 − 5) for many primary tone level combinations. The goal here is to obtain the cancellation parameters (*L*_canc_, *ϕ*_canc_) at *f*_3_ for each pair (*L*_1_, *L*_2_), so that they can later be directly retrieved from the LUT based on the measured primary tone levels.

**Fig. 8:**
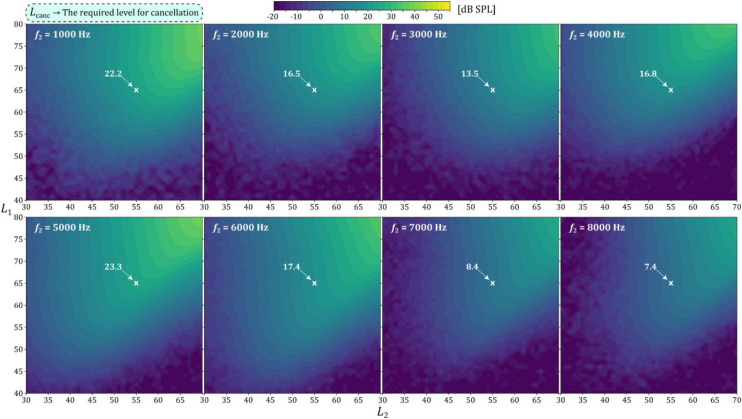
Contour plots of the required cancellation-tone level, *L*_canc_ (dB SPL), as a function of primary levels (*L*_1_, *L*_2_) for different *f*_2_ frequencies. The marker indicates the example operating point (65, 55) dB SPL used in Fig. 7.

**Fig. 9:**
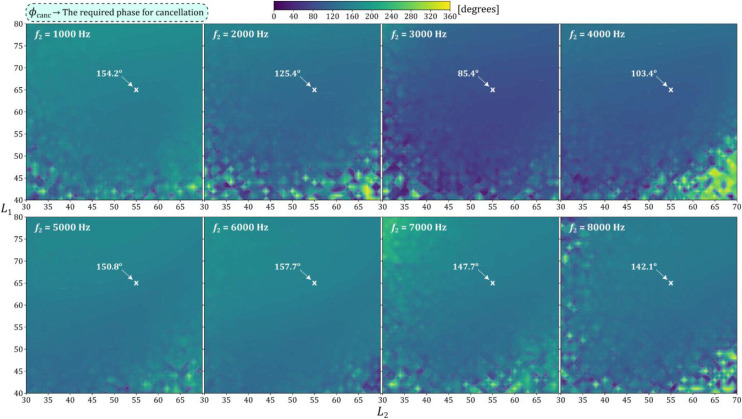
Contour plots of the required cancellation-tone phase *ϕ*_canc_ (degrees), as a function of (*L*_1_, *L*_2_) for different *f*_2_ frequencies. The marker indicates the example operating point (65, 55) dB SPL used in Fig. 7.

First, we defined a range for the primary tone we want to cover (e.g., *L*_1_ = 40–80 dB SPL and *L*_2_ = 30–70 dB SPL). Narrower ranges can also be used. From this range, we constructed a level grid $\left(L_{1}^{\left(i\right)},L_{2}^{\left(j\right)}\right)$. For each point on the grid, we used the same frequency ratio to generate *f*_1_ and *f*_2_ tones (e.g., *f*_2_/*f*_1_ = 1.22). Next, their amplitudes were then set using the frequency-dependent level correction from Step 3, so that the measured level at the probe microphone matched the target $\left(L_{1}^{\left(i\right)},L_{2}^{\left(j\right)}\right)$ in the ear simulator. At each point $\left(L_{1}^{\left(i\right)},L_{2}^{\left(j\right)}\right)$, we ran a two-segment measurement and lock-in analysis (Step 4) to calculate the required cancelling tone level and phase at *f*_3_. The results were $\left(L_{\text{canc}}^{\left(i,j\right)},\phi_{\text{canc}}^{\left(i,j\right)}\right)$. These parameters were then stored as LUT entries, within a 2D array whose indexes follow the tested *L*_1_ and *L*_2_ pairs. Examples of these results are shown in Fig. 8, Fig. 9, which are contour plots of *L*_canc_(*L*_1_, *L*_2_) and *ϕ*_canc_(*L*_1_, *L*_2_) for several test frequencies *f*_2_ (1–8 kHz).

**Fig. 10:**
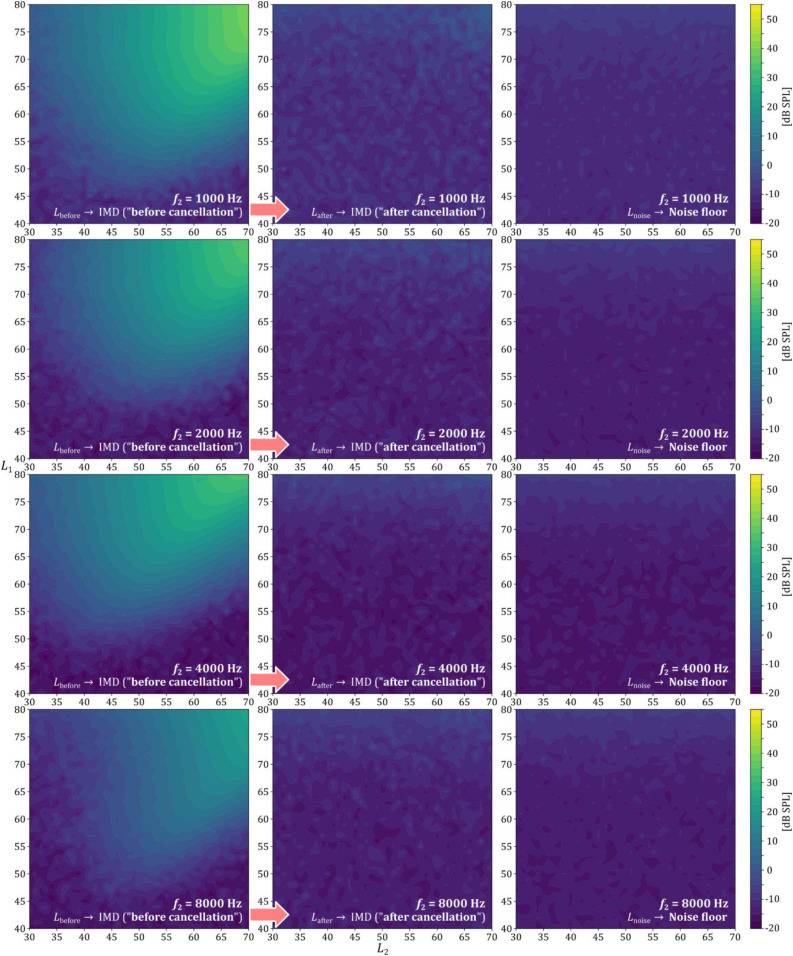
Contour plots of IMD level before cancellation (*L*_before_), residual level after cancellation (*L*_after_), and the local noise-floor estimate (*L*_noise_) across the (*L*_1_, *L*_2_) grid for selected *f*_2_ frequencies.

In addition to storing the LUT entries, we also measured how well the cancellation worked at each point on the grid. To do this, at each $\left(L_{1}^{\left(i\right)},L_{2}^{\left(j\right)}\right)$ we stored three metrics from Step 5: $L_{\text{before}}^{\left(i,j\right)}$ (IMD level before cancellation), $L_{\text{after}}^{\left(i,j\right)}$ (residual level after cancellation), and $L_{\text{noise}}^{\left(i,j\right)}$ (estimated local noise floor). Fig. 10 shows an example of contour plots of these three metrics on the (*L*_1_, *L*_2_) grid for some selected *f*_2_ frequencies. These plots make it easy to check whether the cancellation is working effectively in the desired operating region, and also help identify grid points that need to be remeasured. In our implementation, remeasurement was performed if a grid point was found to be inconsistent with its neighboring points. First, the *L*_canc_ and *ϕ*_canc_ contour maps (Fig. 8, Fig. 9) were examined. These maps typically showed fairly smooth values across the (*L*_1_, *L*_2_) grid. Therefore, if a grid point suddenly stood out in level or phase, the *L*_after_ map (Fig. 10) was reexamined to see if it also showed a high residual IMD value. If so, the point was remeasured. No strict numerical threshold was used for this decision. It was treated as a quality control measure to determine the smoothness of the LUT values around that point. In practice, out of a total of 1681 grid points for a single *f*_2_, only a small number of points required this additional attention, sometimes as few as 10 or 20 points. After remeasurement, these points almost always matched the values of neighboring points. We suspect that this problem was not caused by systematic errors in the cancellation procedure, but rather by occasional instability in the data acquisition, such as possible playback/recording buffer underruns or overruns.

❖ Step 7— *Example use case: applying the LUT to a single ear-canal load*

**Fig. 11:**
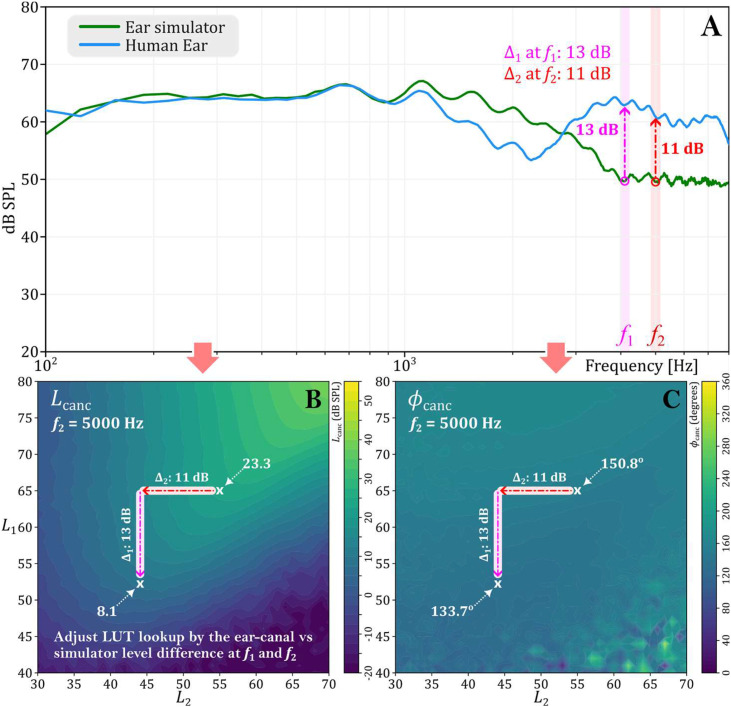
Example of transferring the ear-simulator LUT to an ear-canal load. (A) Earphone–probe frequency responses measured in the ear simulator and in the subject ear canal; the level differences at *f*_1_ and *f*_2_ (Δ_1_, Δ_2_) are used to determine the effective (*L*_1_, *L*_2_) index for LUT lookup. (B) The corresponding shift on the *L*_canc_(*L*_1_, *L*_2_) map and (C) on the *ϕ*_canc_(*L*_1_, *L*_2_) map.

This step provides an example of applying the LUT from an ear simulator to a single human subject (one ear). This example is included to demonstrate how the LUT is used after in-ear calibration, as well as how the cancellation parameters are adjusted to reflect the acoustics of the ear canal. The key point is that the frequency response measurements in the ear canal were performed with the same TSP signal and the same settings as the measurements in the ear simulator in Step 3. Therefore, the difference between the two responses (as seen in Fig. 11A) primarily reflects the difference in acoustic load (ear canal vs. ear simulator), not differences in stimulus.

In this step, the probe assembly (insert earphone + probe microphone) was placed in the ear canal. We then played the TSP through the earphone and recorded it with the probe microphone. Using this recording, the ear canal frequency response *H*_ear_(*f*) was calculated in the same way as the ear simulator response *H*_IEC_(*f*) in Step 3. Based on this *H*_ear_(*f*), we defined the in-ear level correction as *C*_ear_(*f*) = 65 − *H*_ear_(*f*). This correction was used to adjust the primary tones so that the measured levels at *f*_1_ and *f*_2_ in the probe microphone matched the target (e.g., *L*_1_ ≈ 65 dB SPL and *L*_2_ ≈ 55 dB SPL). A comparison of *H*_ear_(*f*) and *H*_IEC_(f) for this subject is shown in Fig. 11A. The next step was to select the “equivalent” cancellation LUT entries in the ear simulator domain. To do this, the response differences at the primary tone frequencies were summarized into two offsets: Δ_1_ at *f*_1_ and Δ_2_ at *f*_2_ (Fig. 11A). The Δ_1_ and Δ_2_ values were then used to determine the effective (*L*_1_, *L*_2_) indices used when reading the ear simulator LUT map. This mapping process is illustrated in Fig. 11B and C for *L*_canc_(*L*_1_, *L*_2_) and *ϕ*_canc_(*L*_1_, *L*_2_). The obtained parameters, (*L*_canc_, *ϕ*_canc_), were then used to generate a cancellation tone at *f*_3_ in the subject’s ear.

**Fig. 12:**
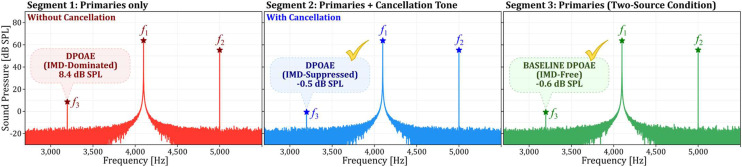
Example in-ear check in one subject showing the DPOAE readout at *f*_3_ for (Segment 1) one-source primaries only (DPOAE masked by IMD), (Segment 2) one-source with LUT-selected cancellation (DPOAE with IMD suppressed), and (Segment 3) two-source baseline (DPOAE, IMD-free reference).

Finally, we performed DPOAE measurement with a three-segment stimulus to verify the results. Segment 1 contained only the primary tones (single source, without cancellation), so that the *f*_3_ component could be contaminated by IMD and mask the DPOAE. Segment 2 contained the same primary tones plus the cancellation tone selected from the LUT (single source, with cancellation), so that the IMD contribution to *f*_3_ could be suppressed. Segment 3 provided a baseline reference measured under two-source conditions (two separate transducers for *f*_1_ and *f*_2_), which is expected to be IMD-free. Fig. 12 shows the results from this measurement. After LUT-based cancellation (Segment 2), the *f*_3_ component (“DPOAE, IMD-suppressed”) is close to the two-source baseline (“DPOAE, IMD-free baseline”), indicating the use of the LUT as intended for a single ear canal.

## Method validation

The validation of the method in this study was conducted to determine two things: whether the IMD cancellation procedure works as intended, and how well it performs under realistic measurement conditions. The most important technical requirement was that the cancellation tone at *f*_3_ must match the IMD generated by the transducer, both in magnitude and phase. Because these two parameters can change when the acoustic load differs from the load used when constructing the LUT (i.e., the ear simulator), the validation was conducted in two complementary stages.

In the first stage (#1), we used a silicone pinna as an ear canal load since it did not generate biological DPOAEs. Therefore, any observed components at *f*_3_ can be attributed to transducer nonlinearity. This stage was used to evaluate how far IMD can be suppressed to near the noise floor, while also demonstrating the difference in results when the cancellation parameters were applied without adjustment and with adjustment of the ear canal transfer response via the LUT. In the second stage (#2), the method was tested on human subjects, where real DPOAEs are present. Performance was evaluated by comparing single-transducer measurements (without cancellation versus with cancellation) against a two-source baseline at various test frequencies.

### Validation in silicone pinnas (no-emission ear-canal load)

#### Dataset, fabrication, and test design

**Fig. 13:**
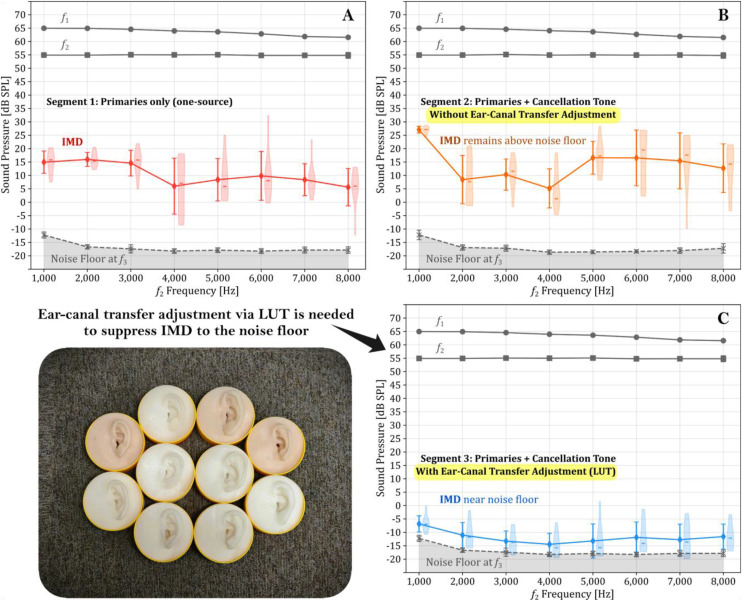
Validation on silicone pinnas showing how ear-canal adjustment via LUT affects IMD suppression at *f*_3_ across *f*_2_ =1–8 kHz. (A) One-source primaries only. (B) Cancellation tone applied using ear-simulator parameters without ear-canal adjustment, leaving IMD above the noise floor. (C) Cancellation tone selected after ear-canal adjustment via the LUT, reducing IMD to near the noise floor. Shaded region indicates the noise floor at *f*_3_. Symbols show mean ± SD across pinnas and violins show the distribution.

To validate IMD suppression of ear canal load without biological emissions, we used silicone pinnas with anatomically realistic ear canals. These ear canal geometries were taken from the IHA database of human geometries, which included 10 individuals [23]. The original data were available as 3D surface models that included the outer ear and the complete ear canal. From this dataset, we extracted the geometry of the left ear for each individual. Then, the mesh was modified so that only the pinna and ear canal were retained for the mold fabrication process. The resulting STL files were exported as pinna-ear canal models for each individual. We included these files in a public repository to allow for replication of this study (https://github.com/iru-one-syah/PinnaModels). Each pinna was then molded with silicone that had a Shore 00 hardness of approximately 40 (measured at room temperature). This hardness value was chosen to approximate the mechanical feel of the human pinna (earlobe) based on our prior measurements of pinna hardness in adults [24]. The model fabrication process (from mesh preparation, mold creation, silicone casting, and finishing) is also provided in a supplementary video (https://youtu.be/WkKg865pGbI). A photograph of the resulting silicone pinnas is shown in the lower left of Fig. 13.

Measurements in the silicone pinnas were performed using the same equipment and analysis flow as in Steps 1–7. For IMD evaluation, we presented three-condition stimulus segments, each lasting 10 s long. Segment 1 (Results in Fig. 13A) presented only the primary tones (single-source) and served as a reference. Because silicone pinnas did not produce any biological emissions (DPOAEs), the component measured at *f*_3_ in this segment represented the IMD originating from the transducer. In Segment 2 (Results in Fig. 13B), we presented the same primary tones and added a cancellation tone at *f*_3_, but without adjustment to the ear canal transfer response. In other words, the level and phase of the cancellation tone were taken directly from the ear simulator LUT at the operating point (*L*_1_, *L*_2_) = (65, 55) dB SPL. In Segment 3 (Results in Fig. 13C), we again presented the same primary tones and cancellation tone at *f*_3_, but this time the cancellation parameters were taken after adjustment to the ear canal transfer response. These parameters were obtained by “translating” the ear simulator LUT to the silicone ear canal load based on the difference between the ear canal transfer response and the ear simulator response, as per Step 7.

#### Validation results

The main purpose of this validation was to verify two practical aspects before moving to DPOAE measurements in humans. First, since silicone pinnas do not produce biological DPOAEs, they can be considered “zero emission.” This allowed us to verify whether the cancellation procedure proposed here was able to suppress the transducer-generated IMD at *f*_3_ to near the noise floor for different ear canals. Second, this validation also tested whether the cancellation parameters obtained in the ear simulator could be directly applied, or whether adjustment of the ear-canal transfer response via the LUT was necessary for effective cancellation. The practical advantage of this adjustment is clearer when considering real-ear level calibration. In our previous study [14], cancellation parameters were obtained at *L*_1_/*L*_2_ = 65/55 dB SPL in an IEC 60318–4 ear simulator and applied to human measurements using the same electrical driver settings. This approach helped demonstrate feasibility. However, in practice, the target primary levels should be achieved in the individual ear canal. For example, in a small ear canal, such as a child’s ear canal, the same electrical drive level that produces 65/55 dB SPL in the ear simulator may produce a higher level, such as 75/65 dB SPL, in the ear canal. To achieve the desired 65/55 dB SPL in that ear, the drive level would need to be reduced by 10 dB in this example. However, after this reduction process, the IMD produced by the transducer will no longer match that measured in the ear simulator under the original driver conditions, and the original cancellation tone level and phase may be inaccurate. Therefore, cancellation parameters should be selected or adjusted after real-ear level calibration. The silicone-pinna validation in Fig. 13 demonstrates this situation.

Fig. 13 shows the results from ten silicone pinnas for *f*_2_ = 1–8 kHz. In Segment 1 (where only primary tones were presented), the IMD was significantly above the noise floor (see Fig. 13A). Also, the clear variability seen in the violin plots indicates that the IMD level depends on the ear canal. In Segment 2 (a cancellation tone was added but without adjustment of the ear-canal transfer response), the IMD still remained above the noise floor (see Fig. 13B). This behavior is expected because the cancellation tone was generated using the ear simulator’s cancellation parameters at the operating point (e.g., *L*_1_, *L*_2_ = 65, 55 dB SPL) without compensating for differences in the frequency response between the silicone ear canal and the ear simulator. As shown in the previous example comparing the ear simulator and the human ear canal in Fig. 11, the same electrical drive level does not always produce the same response at the probe microphone. As a result, the cancellation tone can have a magnitude and phase that does not match the actual IMD. Thus, this mismatch leaves a residual that is not fully canceled and significantly remains above the noise floor.

In Segment 3 (a cancellation tone was added with adjustment of the ear-canal transfer response via the LUT), the residual IMD significantly dropped to near the noise floor at all tested frequencies (Fig. 13C). These results indicate that selecting the cancellation parameters after adjusting the ear-canal transfer response can significantly improve IMD suppression. However, the residual IMD in Fig. 13C does not always fall exactly on the noise floor curve. At most frequencies, it remains slightly above it. Therefore, this residual distortion represents the “practical limit” achievable with the current method. In human measurements, because the biological DPOAE appears at *f*_3_, small residual IMD can, in principle, combine with the DPOAE constructively or destructively, depending on the phase and level differences between the two. Therefore, the results in silicone pinnas should be viewed as confirmation that this method is capable of reducing IMD to near the noise floor in non-emission loads. The subsequent human-subject validation (see the next subsection) is, therefore, needed to determine how the remaining residual IMD compares with typical DPOAE levels and whether it meaningfully biases the measured DPOAEs in humans.

### Validation in human subjects (agreement with baseline)

#### Human subjects and test design

**Fig. 14:**
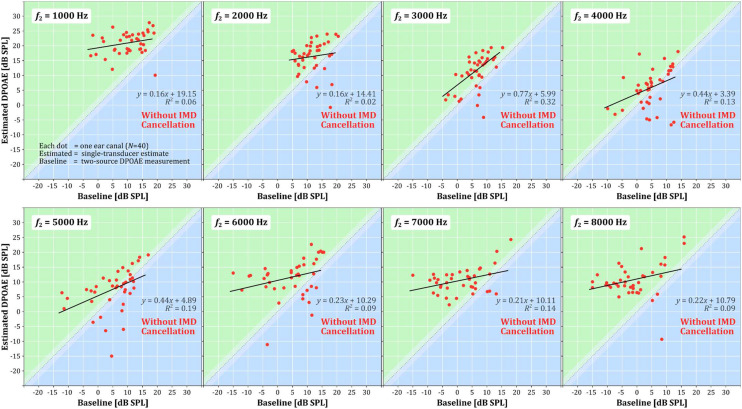
Relationship between baseline DPOAE level and the estimated DPOAE level across test frequencies (*f*_2_ =1–8 kHz) without IMD cancellation. Each dot represents one ear canal (*N* = 40). The diagonal line indicates equality (*y* = *x*), and the solid line shows the linear regression fit. The shaded diagonal band indicates ±2.5 dB from equality.

**Fig. 15:**
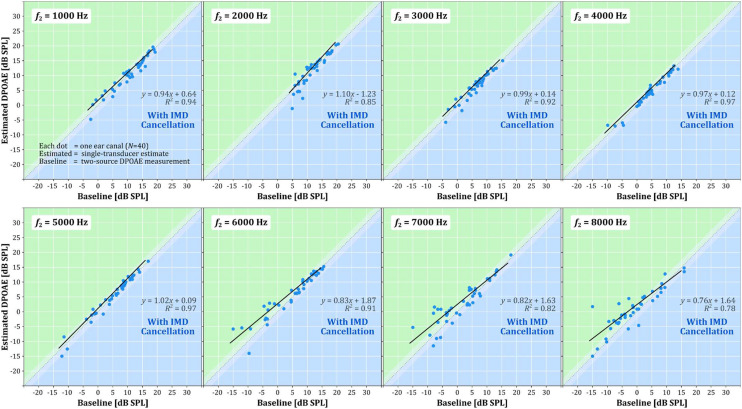
Relationship between baseline DPOAE level and the estimated DPOAE level across test frequencies (*f*_2_ =1–8 kHz) with IMD cancellation. Each dot represents one ear canal (*N* = 40). The diagonal line indicates equality (*y* = *x*), and the solid line shows the linear regression fit. The shaded diagonal band indicates ±2.5 dB from equality.

DPOAE measurements were performed on human subjects to validate the LUT-based IMD cancellation procedure under real ear canals. Additionally, DPOAE results from a single-source condition were compared with a baseline two-source condition. Twenty subjects with normal hearing participated (ages 21–35, mean 23.8 ± 3.2 years; 4 women and 16 men). Measurements were performed on both ears, resulting in a total of 40 ears (ear canals). The measurement procedure followed the example use case in Step 7. The setup used was identical to Table 1 and Fig. 3, and the ear-canal transfer adjustment was also the same. Specifically, the adjustment was performed to “translate” the LUT-based *f*_3_ cancellation tone (amplitude & phase) constructed by the ear simulator so that it could be applied to each subject’s ear canal. The same three-segment stimuli were recorded for each ear. Segment 1 presented the primary tones (single source), Segment 2 presented the primary tones + the LUT-derived cancellation tone (single source), and Segment 3 presented the baseline of the two-source condition. Each segment was 10 s long. The target levels of the primary tones were set to *L*_1_ = 65 dB SPL and *L*_2_ = 55 dB SPL, and the frequency ratio was kept constant at *f*_2_/*f*_1_ = 1.22. DPOAEs at *f*_3_ were extracted using the same FFT-based analysis as in Step 5. The agreement between the single-source and two-source baseline conditions is shown in Fig. 14, Fig. 15, and the RMSE is summarized in Table 2.

**Table 2:** Root-mean-square error (RMSE) between the baseline DPOAE level (two-source measurement) and the estimated DPOAE level (single-transducer estimate) for each test frequency (*f*_2_ = 1–8 kHz). RMSE values are shown without and with IMD cancellation across all ear canals (*N* = 40).

*f*_2_ Frequency [Hz]	1000	2000	3000	4000	5000	6000	7000	8000
RMSE (without cancel. vs baseline) [dB]	**12.06**	**7.72**	**6.65**	**6.78**	**7.65**	**11.19**	**11.44**	**14.49**
RMSE (with cancel. vs baseline) [dB]	**1.41**	**2.02**	**1.21**	**0.97**	**1.23**	**3.06**	**3.54**	**4.65**

#### Validation results

The purpose of this validation on human subjects was to evaluate whether the proposed LUT-based IMD cancellation method can produce consistent DPOAE measurements between the single-source condition and the two-source baseline condition. This comparison was performed under real-ear measurement conditions in 20 subjects (40 ears), where individual ear-canal geometry can differ and ear-specific adjustment was therefore applied. For each test frequency, Fig. 14 shows scatter plots to compare the results from the single-source condition without IMD cancellation against the two-source baseline condition. Fig. 15 also shows the same comparison but for when IMD cancellation is applied. Finally, Table 2 summarizes the overall results for each frequency using root-mean square error (RMSE).

Without IMD cancellation (Fig. 14), most red dots are located primarily in the green area, which indicates that the DPOAE observed in the single-source condition is often higher than the two-source baseline. This pattern matches the example in Fig. 12, where the observed component at *f*_3_ is dominated by IMD rather than the biological DPOAE. As a result, the apparent “DPOAE” is biased upward. A smaller number of red dots also appear well below the diagonal line (i.e., in the blue area, sometimes by >10 dB), which suggests that the observed component at *f*_3_ can also be attenuated in some ears. One possible explanation is destructive interaction between the biological DPOAE and the IMD component at *f*_3_ since the observed component at *f*_3_ depends on their relative magnitude and phase. On the other hand, red dots above the diagonal line can occur when the biological DPOAE and IMD combine constructively, in addition to the simpler case where IMD is much larger than the DPOAE. Overall, the wide spread and frequent bias away from the diagonal line indicate that, without IMD cancellation, the observed components at *f*_3_ in the single-source condition cannot be reliably interpreted as DPOAE levels. This interpretation is supported by the regression results in Fig. 14, where the slopes (*m*: 0.16–0.77) are generally small and the *R*^2^ values (0.02–0.32) are low across frequencies, indicating poor agreement with the two-source baseline.

With IMD cancellation (Fig. 15), agreement with the two-source baseline improves significantly. For *f*_2_ = 1–5 kHz, most blue dots cluster around the diagonal line, and the fitted slopes are near unity (*m*: 0.94–1.10). This is consistent with effective IMD cancellation at *f*_3_, so that the remaining component reflects the biological DPOAE. At *f*_2_ = 6–8 kHz, agreement is still better than without cancellation, but performance decreases (*m*: 0.76–0.83). The spread increases, and deviations become more frequent, especially when the baseline DPOAE is below 0 dB SPL. One possible explanation is residual IMD that does not always fall to the noise floor (as also suggested by the silicone-pinna validation), which can bias the single-source estimate when the biological DPOAE is small, depending on phase. The regression lines and *R*^2^ values in Fig. 15 support this trend, showing strong correspondence at 1–5 kHz and reduced agreement at 6–8 kHz. Finally, Table 2 summarizes the overall results. Without IMD cancellation, RMSE is large across frequencies (6.65–14.49 dB), consistent with the wide spread in Fig. 14. With IMD cancellation, RMSE drops to 0.97–4.65 dB, confirming improved agreement with the two-source baseline. Errors are smallest at 1–5 kHz (≤2 dB) and remain larger at 6–8 kHz (3.06–4.65 dB), consistent with the increased spread in Fig. 15 when baseline DPOAE levels are low. The reduction in performance at high frequencies is probably due to IMD cancellation becoming more sensitive to small changes in probe position. To cancel out IMD at *f*_3_, the tone must be at the same level as the IMD at *f*_3_ and almost opposite in phase. But small shifts in probe position can change the acoustic path from the earphone to the probe microphone. At low frequencies, these shifts in position are a small fraction of a wavelength and so produce only small changes in phase. At high frequencies, the wavelength is shorter. That means the same probe movement results in a bigger phase shift. Consequently, cancellation parameters that are effective at low frequencies can become mismatched at higher frequencies, leaving some residual IMD. Furthermore, DPOAE levels at baseline in the 6–8 kHz range were often lower than those at low frequencies. When biological DPOAE is low, even small residual IMD can strongly influence measurements through constructive or destructive interactions. These two factors may help explain why the agreement with the two-source baseline decreased at higher frequencies. In general, our validation in human ears shows that the LUT-based cancellation method improves agreement with a two-source baseline by suppressing IMD at *f*_3_ using standard probe hardware.

### Interpretation and clinical implications of residual IMD

It is important to interpret the difference between results from silicone pinnas and human ears with some caution. There is no biological DPOAE in silicone pinnas, so any residual component at *f*_3_ after cancellation is directly observable as residual IMD. But in human ears, the measured *f*_3_ component contains the biological DPOAE. Thus, when the DPOAE level is sufficiently above the residual IMD, as often seen at 1–5 kHz in Fig. 15, the agreement with the two-source baseline may look better when in fact a small residual IMD may still be present. This is mainly because biological DPOAEs are present in human ears but absent in silicone pinnas. One other factor is the lack of a realistic tympanic membrane and middle-ear termination in silicone pinnas, which might affect the acoustic load. Still, this was not considered the main reason for the observed differences.

From a clinical perspective, the residual IMD shown in Fig. 10, Fig. 13 should also be considered when interpreting the human-subject results in Fig. 15. Here, we consider the implications of residual IMD for clinical pass/fail screening, where the presence or absence of a reliable DPOAE response is used as part of the decision criterion. In this study, the human-subject validation was performed in normal-hearing ears, where measurable DPOAEs were generally present. Under these conditions, the impact of small residual IMD may be limited when the biological DPOAE level is sufficiently higher than the residual artifact (as observed at 1–5 kHz in Fig. 15). However, this problem becomes more serious in ears with hearing loss, where DPOAEs may be weak, or in deaf ears, where biological DPOAEs are not expected. In these cases, residual IMD may be misinterpreted as a true DPOAE response, increasing the risk of false-pass results (i.e., DPOAE detected despite reduced or absent cochlear amplification). Therefore, additional validation in ears with reduced or absent DPOAEs is necessary before using this method for clinical pass/fail screening. In the current implementation, this concern is also particularly relevant at frequencies of 6 kHz and above, where the residual IMD after cancellation may still fall within the level range of low high-frequency DPOAEs. Therefore, pass/fail decisions should not rely mainly on these high-frequency results until further validation is performed. Conservative criteria, such as a minimum acceptable DPOAE level or signal-to-noise ratio, may reduce false-positive detection due to residual IMD, but may also reduce sensitivity to weak biological DPOAEs.

## Limitations

One limitation of our proposed method is the time needed to fill the LUT. For example, if *L*_1_ is set to 40 to 80 dB SPL and *L*_2_ is set to 30 to 70 dB SPL with 1 dB increments, the number of grid points per *f*_2_ is 41 × 41 = 1681. So, repeating this process for *f*_2_ = 1 to 8 kHz (1 kHz increments, 8 frequencies) results in a total of 13,448 points, each of which requires at least (i) an estimation run and (ii) a verification run. As a result, even if we try to keep each measurement segment short, creating a complete LUT can take hours. This burden, in practice, can be reduced by limiting the grid to the expected operating range (such as ±10 dB of the target *L*_1_/*L*_2_), using coarse step sizes with interpolation, or using adaptive sampling to measure densely only in areas where *L*_canc_ and *ϕ*_canc_ change sharply and sparsely in areas where they change smoothly. This also means that the method is less flexible than a conventional two-transducer probe when many arbitrary frequency and level combinations are required. Therefore, for research applications that require broad stimulus flexibility, a two-transducer probe may still be more practical. However, the LUT burden becomes more manageable when the measurement protocol is restricted to selected DPOAE conditions, such as a limited frequency range, equal-level conditions, a fixed (*L*_1_ − *L*_2_) offset, or a scissor paradigm. In such cases, only the required frequency and level combinations need to be included in the LUT, which may make the approach practical for compact or low-cost single-transducer devices. Finally, even after IMD suppression, a residual IMD can still remain and bias the actual DPOAE via constructive or destructive interactions, which could affect the accuracy of the results. This residual may also be difficult to evaluate when it is close to the noise floor, because the verification spectra in this study were obtained from a single FFT of the full 3-s analysis window, without integer-period frequency adjustment or time-domain averaging. A possible improvement would be to divide the 3-s window into thirty 100-ms intervals, adjust the stimulus frequencies so that each interval contains an integer number of periods, and then apply coherent time-domain averaging before FFT analysis. This would provide the coherent-averaging gain of up to 10log_10_(30), or about 14.8 dB, for uncorrelated noise and may make small residual IMD components easier to detect.

Another practical limitation relates to the reusability of the LUT between different transducers. In this study, we built and validated the LUT using the same insert earphone and probe microphone configuration. While the ear-specific adjustment described in this method accounts for differences between ear simulators and individual ear canals, it cannot be assumed to fully compensate for differences between transducer units. Differences in overall sensitivity can be partially addressed by SPL calibration and level correction, but differences in the generation of nonlinear IMD can alter the required level and phase cancellation. Therefore, when using different transducer units, the LUT should be rebuilt or at least validated before use. This requirement can reduce the practicality of this method when many units are used. This highlights the need for a more general strategy that relies less on device-specific calibration.

In DPOAE research, other stimulus patterns, such as swept tones, chirps, or stimulus pulses, are also used. This is because they can support component separation using least squares methods and time-domain analysis. In principle, while it may be possible to apply the present method to sweep tone- or chirp-based measurements, a modified calibration procedure would be required. Since the distortion frequency (*f*_3_ = 2*f*_1_ – *f*_2_) changes continuously with changes in *f*_1_ and *f*_2_, in that case, the cancellation signal would need to have time-varying level and phase, and the method for estimating these parameters would need to be newly defined. On the other hand, short stimulus pulses would present an additional challenge, since the generated distortion would no longer appear as a single steady component at *f*_3_, but rather as a transient and broadband response. For this reason, the present method should be considered specific to steady-state pure-tone DPOAE measurements.

## Related research article for a published article

None.

## Ethics statements

This study involving human participants was approved by the Institutional Review Board of Life Science Research, Chiba University. All participants provided written informed consent before taking part in this study.

## CRediT authorship contribution statement

**Irwansyah:** Conceptualization, Methodology, Software, Visualization, Investigation, Writing – original draft, Writing – review & editing. **Sho Otsuka:** Formal analysis, Writing – review & editing. **Seiji Nakagawa:** Formal analysis, Writing – review & editing, Supervision, Funding acquisition, Resources.

## Declaration of competing interest

The authors declare that they have no known competing financial interests or personal relationships that could have appeared to influence the work reported in this paper.

## Data Availability

Data will be made available on request.
